# Growing pains: contemporary knowledge and recommended practice

**DOI:** 10.1186/1757-1146-1-4

**Published:** 2008-07-28

**Authors:** Angela M Evans

**Affiliations:** 1 School of Health Science, Division of Health Science, University of South Australia, City East Campus, North Terrace, Adelaide, 5000, Australia

## Abstract

**Background:**

Leg pain in children, described as *growing pains*, is a frequent clinical presentation seen by an array of health care professionals. Described since 1823, growing pains continues to puzzle practitioners, yet diagnostic criteria and evidence based treatment is available.

**Methods:**

The medical literature has been searched exhaustively to access all articles (English language) pertaining to leg pains in children which are ascribed to being 'growing pains'.

**Results:**

The literature, whilst plentiful in quantity and spanning two centuries, is generally replete with reiterated opinion and anecdote and lacking in scientific rigour. The author searched 45 articles for relevance, determined according to title, abstract and full text, resulting in a yield of 22 original studies and 23 review articles. From the original studies, one small (non-blinded) randomised controlled trial that focused on GP treatment with leg muscle stretching was found. Nine prevalence studies were found revealing disparate estimates. Ten cohort (some case-controlled) studies, which investigated pain attribute differences in affected versus unaffected groups, were found. One series of single case experiment designs and one animal model study were found.

**Conclusion:**

Growing pains is prevalent in young children, presents frequently in the health care setting where it is poorly managed and is continuing to be researched. A common childhood complaint, growing pains needs to be acknowledged and better managed in the contemporary medical setting.

## Background

Growing pains first appeared as a described entity in the medical literature in 1823 following the observations of a French physician Marcel Duchamp [1]. Although the topic of many reports since that time [2-11], and despite being a frequent paediatric clinical presentation, growing pains remains largely misunderstood [12-14] and as a result poorly managed [2,15]. The purpose of this article is to compile a contemporary summary of what is known about growing pains and to provide a management guideline from the currently available scientific evidence.

## Methods

The medical literature has been searched exhaustively to access all articles (English language) pertaining to leg pains in children which are ascribed to being 'growing pains'. It is important to note however, that growing pains (defined in Table 1) are not the same as all non-specific leg pains. Many previous reviews have utilised the literature conveniently rather than comprehensively which has resulted in many incomplete and misleading articles amid the body of knowledge [16].

**Table 1:** Definition of 'growing pains' – inclusion and exclusion criteria.

**Pain factors**	**Inclusion criteria**	**Exclusion criteria**
Nature of pain	Intermittent Some pain free days and nights	Persistent Increasing intensity
Unilateral or bilateral	Bilateral	Unilateral
Location of pain	Anterior thigh, calf, posterior knee – in muscles	Joint pain
Onset of pain	Late afternoon or evening	Pain still present next morning
Physical examination	Normal	Swelling, erythema, tenderness Local trauma or infection Reduced joint range of motion Limping
Laboratory tests	Normal	Objective findings eg ESR, x-ray, bone scan abnormalities
Limitation of activity	Nil	Reduced physical activity

The subject search used a combination of controlled vocabulary, MeSH headings and free text terms based on the following search strategy for searching MEDLINE:

# 1 growing pain* or leg pain* or leg ache*

# 2 paediatric or pediatric or child*

# 3 #1 and #2

The electronic databases searched were:

1. Cochrane Pain, Palliative & Supportive Care Register (current issue)

2. The Cochrane Controlled Trials Register: Cochrane Library (current issue)

3. MEDLINE (1966 – present)

4. EMBASE (1980 – present)

5. CINAHL (1960 – present)

6. AMI (- present)

7. AMED (1985 – present)

8. Current Contents (1993 – present)

In addition, the reference lists of all eligible trials, key textbooks, and previous reviews were searched for additional studies.

The literature presents with recurring themes which have formed the basis for the structure of this present review. In this review, growing pains will be discussed under the following five sub-headings which reflect the body of knowledge found within the scientific literature: definition, prevalence, aetiology, associations and treatment.

## Results

### Definition

There is no single diagnostic test for growing pains and as a result it continues to be diagnosed on the basis of both inclusion and exclusion criteria [2,15,17,18] (Table 1). Misdiagnoses of children with less common but potentially more serious conditions including rheumatoid arthritis (articular pain) or bone tumours (unlikely to be bilateral and night time occurrence) are unlikely if these criteria are adhered to and can be investigated further with blood analyses and imaging if suspected. A recent matched case-control study concluded that growing pain remains a clinical diagnosis and if precise inclusion and exclusion criteria are considered, there is no need for laboratory tests to make a diagnosis [19].

### Prevalence

Studies of the prevalence of growing pains have presented a wide range of estimates from 2.6 to 49.4% [8,11,14,20-23]. Poor sampling, disparate age ranges and non-defined, variable criteria account for much of this latitude. A robust prevalence study established the prevalence of growing pains in children aged four to six years as 37% [24].

### Aetiology

Growing pains remains enigmatic in terms of its cause. Three main theories have been traditionally proposed as follows:

#### Anatomical

The anatomical theory emerged in the 1950's when the previously suspected association between growing pains and rheumatic fever had been overthrown [25]. Scoliosis, lordosis, genu valgum and flat feet have all been cited but unsubstantiated associations [20]. The anatomical theory centred on the premise that a cause of the pain was a postural or an orthopaedic defect that could induce bad posture or stance and that treatment of these 'defects' were clinically observed to give relief. The anatomical theory has recently been weakened with the research findings that foot posture and growing pains are uncorrelated [26].

#### Fatigue

The notion of muscular fatigue as the cause of growing pains was initiated by Bennie in 1894 from clinical case observations [4]. This theory has been periodically reiterated, focussing on a surmised accumulation of metabolic waste products within the leg muscles, but remains untested [21,27,28]. Parents will often associate episodes of growing pains with periods of increased physical activity [15].

#### Psychological

The emotional or psychological theory was introduced in 1951 [21] and has been further cited and addressed as possible causative factor by many authors since [2,22,28,29]. Increased vulnerability to pain has been suspected as has a familial predisposition. There is dissent regarding gender bias, where girls have historically been regarded as more susceptible [22]. Oberklaid investigated children with growing pains as part of a wider temperament survey and found that parents of affected children rated them to have a negative or intense mood [23].

#### Further theories of pathogenesis

Many investigations into the cause of growing pains have ensued in the last decade. Indeed, it is notable that this condition has continued to captivate clinicians and researchers with 185 years of reported history within the medical literature. Table 2 summarises the four recent studies which have developed new theory for the aetiology of growing pains, as referred to in the following text:

**Table 2:** Summary of the recent studies which have established new aetiological theory for growing pains (GP).

**Date**	**First Author**	**Sample** ** size**	**Research design**	**Findings**	**New theory**
2004	Hashkes, PJ	GP group: n = 44 No GP control: n = 46	Case control Dolorimeter (pressure)	GP group had lower pain thresholds	GP may be a variant of a non-inflammatory pain syndrome
2005	Friedland, O	GP group: n = 39 No GP control: n =	Case control Ultrasound bone speed, tibia and radius	GP group had reduced tibial bone speed.	GP may represent a local overuse syndrome.
2005	Hashkes, PJ	GP group: n = 11 No GP control: n = 12	Case control Bone scintigraphy, tibia	GP group did not have altered vascular perfusion when compared with control group	GP are not associated with altered vascular perfusion as opposed to migraine

(i) Lower pain threshold: The pain threshold in children with growing pains has been found to be significantly reduced in comparison to an age and gender matched control group [30]. The authors suggest this may indicate that growing pains is a generalised non-inflammatory pain syndrome occurring in childhood.

(ii) Decreased bone strength: The speed of sound through bone was assessed using ultrasound and it was found that the bone strength density of the tibia in children with growing pains was significantly less than for normative data [31]. The authors postulate that bone fatigue with activity may give rise to the leg pains.

(iii) Altered vascular perfusion: Investigation of the uptake of technetium-99 during bone scans has been found not to differ in small samples of children with growing pains versus unmatched controls [32]. The authors hence refuted the hypothesis that growing pains may be induced by altered vascular perfusion in a manner similar to migraine headaches.

(iv) Joint hypermobility: There is untested clinical impression that children with growing pains may be hypermobile similarly to children with fibromyalgia [33,34]. As there is no universally reliable and valid assessment tool for hypermobility in children, support for this notion remains pending [35].

#### Associations

(i) The profile of affected children and the frequency of pain episodes has been recently reported [15]. Children with growing pains were found to be approximately 5% heavier, but not taller than children not reporting growing pains. A positive family history of growing pains was reported, with affected children having either a parent or sibling having experienced growing pains in almost 70% of cases. Most children were reported to experience growing pains in spates with frequency of one to three months [15].

(ii) Previous studies have associated growing pains with abdominal pain, headache, as part of a pain triad [29,36] an area which is still somewhat unclear.

(iii) Growth has been associated [1,5,27,37-39] and disassociated [2,12,13] with growing pains, but little investigation has ensued. Childhood is, by definition, a time of growth, but growth per se as a source of pain is uncertain and contentious [40,41]. Preliminary results found recumbent posture to be associated with increased tibial growth in three lambs [37]. Clearly this preliminary finding cannot be validly transposed to human subjects. Parents of children with growing pains associated growing pains and increased growth in 35% of cases [15].

(iv) Increased levels of lead, zinc and decreased levels of copper and magnesium have been detected in the hair of children with growing pains, but the usefulness of the analysis of elements in hair remains controversial and has yet to be validated [42].

(v) Flatfeet have been postulated as an aetiological factor for growing pains for many years [20] with preliminary support from single case experiments [43]. A recent comparative study has however, found no clinically significant difference in the foot posture of children with or without growing pains [26].

(vi) Increased activity levels have been found to be associated by the parents of children with growing pains in 37% of reports [15]. Opinions over many years lend support to this preliminary finding [4,23,34,39,44].

(vii) Children's quality of life (QoL) when affected with growing pains has been little investigated, despite being such a frequent clinical presentation [2,45]. It has been reported as a preliminary finding that parents assessed reduction in their child's QoL due to growing pains in some 5% of cases [15].

### Treatment

There is only one randomised controlled trial which offers evidence for the treatment of children with growing pains, summarised in Table 3[46]. This small, non-blinded trial offers best (if limited) evidence for the management of growing pains with muscle stretching. Despite this being the best available evidence, it is not dispensed by health professionals who when infrequently consulted (only 34% of children were seen by health professionals [15]) dispense paracetamol. In addition, parents practice the time-honoured methods of rubbing children's legs and using hot water bottles during periods of distress [15].

**Table 3:** Summary of the only randomised controlled trial for treatment of growing pains (GP) (Baxter & Dulberg, 1988).

**No. pain episodes per month**	**Group 1 – treatment** Muscle stretching program * n = 18	**Group 2 – control** **Reassurance, leg rubs, acetyl-salicylic acid** **n = 16**
Beginning of trial	10	10
3 months	1	6
9 months	0	3
18 months	0	2

The RCT for management of GP revealed a statistically significant difference between the treatment and control groups of children (aged 5 – 14 years). However the study was biased, with no examiner blinding. Additionally, sample sizes are small and statistical power was not calculated.

* Parents were taught a muscle stretching program for quadriceps, hamstrings and gastroc-soleal groups. All stretches were performed twice daily (morning and evening) for 10 minutes each time.

Much lower on the evidence hierarchy, single case experiments supported the use of in-shoe wedges and foot orthoses [43]. In addition to the frequently practiced parental methods of treatment using paracetamol, leg rubs and heat, the literature is replete with many unfounded treatments including: vitamin C, D, magnesium, calcium, reassurance [34]. Clearly the first line treatment for growing pains should be that supported by (best available randomised controlled trial) evidence in the form of a muscle stretching program for the quadricep, hamstring and tricep surae groups [46]. Only once muscle stretching has been instituted should any supplementary treatments be appended, if needed.

## Conclusion

Much has been written about growing pains over many years. In common with numerous medical conditions, there is much opinion and a relative paucity of sound science to guide clinicians. That being said, the last decade has seen some clarity and with confidence the contemporary clinician and researcher can be assuaged of the following tenets:

(i) Growing pains is prevalent in children aged four to six years (37%)

(ii) The diagnosis of growing pains is made clinically utilising both inclusion and exclusion criteria

(iii) Growing pains is familial

(iv) Growing pains is not associated with flat feet

(v) Health professionals are usually not consulted and the most practiced methods of management are paracetamol, rubbing legs and heat packs

(vi) The best evidence for management is muscle stretching of quadriceps, hamstrings and triceps surae groups

Contemporary practice should be informed an influenced by this current summary and by future research into this prevalent and frequently presenting childhood complaint.

## Competing interests

The author declares that she has no competing interests.
